# Mast Cell-Specific Deletion of Group III Secreted Phospholipase A_2_ Impairs Mast Cell Maturation and Functions

**DOI:** 10.3390/cells10071691

**Published:** 2021-07-04

**Authors:** Yoshitaka Taketomi, Yuki Endo, Takayoshi Higashi, Remi Murase, Tomio Ono, Choji Taya, Tetsuyuki Kobayashi, Makoto Murakami

**Affiliations:** 1 Center for Disease Biology and integrative Medicine, Laboratory of Microenvironmental and Metabolic Health Science, Graduate School of Medicine, The University of Tokyo, 7-3-1 Hongo, Bunkyo-ku, Tokyo 113-8655, Japan; taketomiys@m.u-tokyo.ac.jp (Y.T.); tahigashi@m.u-tokyo.ac.jp (T.H.); 2 Lipid Metabolism Project, Tokyo Metropolitan Institute of Medical Science, 2-1-6 Kamikitazawa, Setagaya-ku, Tokyo 156-8506, Japan; y.endo.g.c@gmail.com (Y.E.); r_murase@pharm.showa-u.ac.jp (R.M.); 3 Department of Biology, Faculty of Science, Ochanomizu University, 2-1-1 Otsuka, Bunkyo-ku, Tokyo 112-8610, Japan; kobayashi.tetsuyuki@ocha.ac.jp; 4 Center for Basic Technology Research, Tokyo Metropolitan Institute of Medical Science, 2-1-6 Kamikitazawa, Setagaya-ku, Tokyo 156-8506, Japan; ono-tm@igakuken.or.jp (T.O.); taya-cj@igakuken.or.jp (C.T.)

**Keywords:** mast cells, phospholipase A_2_, lipid mediator, anaphylaxis, contact dermatitis

## Abstract

Tissue-resident mast cells (MCs) have important roles in IgE-associated and -independent allergic reactions. Although microenvironmental alterations in MC phenotypes affect the susceptibility to allergy, understanding of the regulation of MC maturation is still incomplete. We previously reported that group III secreted phospholipase A_2_ (sPLA_2_-III) released from immature MCs is functionally coupled with lipocalin-type prostaglandin D_2_ (PGD_2_) synthase in neighboring fibroblasts to supply a microenvironmental pool of PGD_2_, which in turn acts on the PGD_2_ receptor DP1 on MCs to promote their proper maturation. In the present study, we reevaluated the role of sPLA_2_-III in MCs using a newly generated MC-specific *Pla2g3*-deficient mouse strain. Mice lacking sPLA_2_-III specifically in MCs, like those lacking the enzyme in all tissues, had immature MCs and displayed reduced local and systemic anaphylactic responses. Furthermore, MC-specific *Pla2g3*-deficient mice, as well as MC-deficient *Kit*^W-sh^ mice reconstituted with MCs prepared from global *Pla2g3-*null mice, displayed a significant reduction in irritant contact dermatitis (ICD) and an aggravation of contact hypersensitivity (CHS). The increased CHS response by *Pla2g3* deficiency depended at least partly on the reduced expression of hematopoietic PGD_2_ synthase and thereby reduced production of PGD_2_ due to immaturity of MCs. Overall, our present study has confirmed that MC-secreted sPLA_2_-III promotes MC maturation, thereby facilitating acute anaphylactic and ICD reactions and limiting delayed CHS response.

## 1. Introduction

Mast cells (MCs) promote acute allergic reactions, including anaphylaxis, a severe and potentially fatal immunoglobulin (IgE)-dependent immediate hypersensitivity reaction to apparently harmless antigen (Ag), as well as certain IgE-independent innate and adaptive immune disorders [1,2,3]. In addition to these detrimental functions, MCs also have beneficial functions by regulating both innate and adaptive immune responses against invading microorganisms, venom, and environmental toxins [1,2,3]. Crosslinking of the high-affinity receptor for IgE (FcεRI) on the surface of MCs with IgE and specific Ag initiates signals leading to the release of preformed, granule-stored molecules (degranulation), such as histamine and proteases, newly synthesized lipid mediators, such as the arachidonic acid (AA) metabolites prostaglandin D_2_ (PGD_2_) and leukotriene C_4_, and prestored or newly transcribed cytokines and chemokines such as tumor necrosis factor-α (TNF-α) and CCL2, all of which can be involved in the IgE-associated diseases [2,4,5]. The secretion of these bioactive factors by MCs can also be directly activated by diverse stimuli independently of IgE, and many of these factors are known to be present locally at sites of allergic inflammation [3,6,7].

Microenvironment alterations in MC phenotypes affect the susceptibility to hypersensitivity reactions [8]. MCs are derived from progenitors that undergo their terminal maturation after migrating into most vascularized tissues [5,8,9,10]. Based on recent studies, at least two different pathways for MC development have been described in mice; MCs in several connective tissues, such as the skin and peritoneum, originate from yolk-sac-derived MC progenitors at the embryonic stage [11,12], while MCs in other tissues arise from classical hematopoietic stem cells in the bone marrow (BM), with precursors traveling through the circulation before they acquire more mature characteristics in peripheral tissues [10,13,14,15]. The local development and maturation of MCs are thought to be regulated by signals provided by stromal cells within local tissue microenvironments. It is well known that the stem cell factor (SCF) and its receptor c-Kit (CD117) system, in cooperation with several transcription factors, is essential for adhesion, homing, proliferation, and differentiation of MCs [14]. However, as SCF alone is insufficient to fully drive the terminal maturation of MCs, it has been hypothesized that some other stromal factor(s) may be additionally required. Besides several accessory cytokines, growth factors, adhesion molecules, and extracellular matrices as potential candidates for these stromal factors [8,10,15], we have recently shown that a signal driven by the lipid mediator PGD_2_ represents a missing link required for the fibroblast-driven maturation of MCs [16].

The production of PGD_2_ is initiated by hydrolysis of membrane phospholipids by phospholipase A_2_ (PLA_2_). Of many PLA_2_ enzymes identified to date [17,18], group IVA cytosolic PLA_2_ (cPLA_2_α) is essential for the stimulus-coupled release of AA and subsequent production of PGD_2_ by hematopoietic PGD_2_ synthase (H-PGDS) in MCs [19,20,21]. The MC-derived, cPLA_2_α/H-PGDS-driven PGD_2_ exacerbates or attenuates allergic responses by acting on either of the two PGD_2_ receptors DP1 and DP2 (also known as CRTH2) expressed on different target cells [16,22,23,24,25,26,27]. In addition to this MC-intrinsic production of PGD_2_, MCs also regulate the production of a distinct pool of PGD_2_ by stromal fibroblasts, a process that is mediated by the paracrine action of secreted PLA_2_ (sPLA_2_) [16]. The sPLA_2_ family, which contains 11 isoforms in mammals, is structurally subdivided into group I/II/V/X, group III, and group XII branches [28]. Importantly, we have demonstrated that group III sPLA_2_ (sPLA_2_-III; encoded by *Pla2g3*), which is structurally similar to bee venom sPLA_2_ [29], is secreted from immature MCs and functionally coupled with lipocalin-type PGDS (L-PGDS) in neighboring fibroblasts as a paracrine factor to supply a microenvironmental pool of PGD_2_, which in turn acts on the PGD_2_ receptor DP1 on MCs to coordinate proper MC maturation [16]. Accordingly, mice lacking sPLA_2_-III, as well as those lacking L-PGDS or DP1, have immature MCs and display reduced local and systemic anaphylaxis in response to IgE-dependent and -independent stimuli. In addition, the defective MC maturation by *Pla2g3* deficiency eventually leads to impaired cPLA_2_α/H-PGDS-driven PGD_2_ generation by MCs, implying that sPLA_2_-III drives both PGD_2_ pools in direct and indirect fashions in the context of MC–fibroblast interaction.

The contribution of MC-derived sPLA_2_-III to MC maturation was supported by the observation that the engraftment of BM-derived mast cells (BMMCs; an immature population of MCs) prepared from global *Pla2g3*-deficient (*Pla2g3*^−/−^) mice into *Kit^W-sh^* mice, which are intrinsically devoid of MCs due to a mutation in the SCF receptor Kit [30], resulted in defective MC maturation [16]. However, since *Kit*-mutant mice have several phenotypic abnormalities in addition to their MC deficiency [30,31] and since adoptively transferred BMMCs into *Kit*-mutant mice may not be fully identical (in terms of anatomical location, phenotype, or function) to those in the same anatomical location as in the corresponding wild-type (WT) mice [32,33], the use “MC-specific Cre” mice, which enable us to manipulate a target gene only in MCs, has recently been appreciated. In this study, in order to confirm the role of sPLA_2_-III expressed in MCs but not in any other cell types that potentially express this enzyme, we generated MC-specific *Pla2g3* knockout (KO) mice by crossing *Pla2g3*-floxed (*Pla2g3*^fl/fl^) mice with *Mcpt5-Cre* mice, which have been used for MC-specific deletion of target genes [34,35,36]. We provide evidence that MC-specific *Pla2g3* deficiency fully recapitulates the MC maturation defects as observed in global *Pla2g3* deficiency. Additionally, by using MC-specific *Pla2g3* KO mice as well as *Kit^W-sh^* mice adoptively transferred with *Pla2g3*^−/−^ BMMCs, we addressed the roles of MC-derived sPLA_2_-III in irritant contact dermatitis (ICD), an acute inflammation, and contact hypersensitivity (CHS), a Th1-dependent delayed-type allergic response.

## 2. Materials and Methods

### 2.1. Mice

The targeting vector for the *Pla2g3* gene was obtained from the Knockout Mouse Project (KOMP) Repository (clone PRPGS00113_A_H09, Project No. 33384; The Knockout Mouse Project, Mouse Biology Program, University of California, Davis, CA, USA; www.KOMP.org (accessed on 4 April 2011)) [37]. The *Pla2g3* conditional-ready mutant allele was generated by KOMP through insertion of the promoter-driven L1L2_Bact_P cassette into the mouse *Pla2g3* gene at chromosome 11. The functional gene product is composed of 2 *FRT* sites flanking an *IRES*:*lacZ*-trapping cassette and a floxed human β-actin promoter-driven *neo* cassette inserted into the intron 1 of the *Pla2g3* gene and an additional third *loxP* site downstream of exon 3. The targeting vector was transferred into C57BL/6 mouse-derived embryonic stem (ES) cells (RENKA; Niigata University, Niigata, Japan) [38] via electroporation, and the cells that contained the correctly targeted *Pla2g3* locus were identified by PCR and confirmed by Southern blot analysis. Chimeric mice were generated with the recombinant ES cells using an aggregation method. Two chimeras with higher than ~70% coat color chimerism were mated with C57BL/6N mice (Japan SLC, Shizuoka, Japan) to achieve germline transmission. We then generated a conditional KO mouse strain by crossing heterozygous mice with *CAG*-*Flpe* mice (The Jackson Laboratory, Bar Harbor, ME, USA) expressing the flippase recombinase under the control of the actin promoter. This resulted in the excision of the *IRES*:*lacZ* and *neo* cassettes and the generation of a floxed allele. We further crossed conditional KO mice with *Mcpt5*-*Cre* mice expressing the Cre recombinase under the control of the MC-specific protease *Mcpt5* promoter, generously provided by Dr. Axel Roers (Institute for Immunology, Medical Facility Carl Gustav Carus, University of Technology Dresden, Dresden, Germany) [39], to obtain a null allele. MC-specific *Pla2g3* KO (*Pla2g3*^fl/fl^*Mcpt5*-*Cre*) mice and control littermates (*Pla2g3*^fl/fl^) were used for all animal experiments. Mouse genotypes were determined by PCR of tail-snip DNA using GeneAmp Fast PCR Master Mix (Thermo Fisher Scientific-Applied Biosystems, Waltham, MA, USA) and genotyping primers as follows: forward, 5′-GCGCCATTGCTCGAACTGTGGTTG-3′; reverse, 5′-AGGCCAGGCACAGTCTTTCCTCT-3′.

Global *Pla2g3*^−/−^ mice were described previously [16,40]. *Hpgds*^−/−^ mice [41] were provided by Dr. Yoshihiro Urade (Osaka Bioscience Institute, Osaka, Japan). MC-deficient *Kit*^W-sh^ mice (*Kit*^W-sh^HNihrLaeBsmJ, Stock No. 005051) were purchased from the Jackson Laboratory. Age-matched male mice (8–12 weeks of age) were used in each experiment. Mice were maintained in animal facilities in the Tokyo Metropolitan Institute of Medical Science and the University of Tokyo under specific pathogen-free conditions. All animal experiments were approved by the institution and conformed to the Japanese Guide for the Care and Use of Laboratory Animals.

### 2.2. Maturation and Activation of BMMCs

Mouse BM cells were cultured in Dulbecco’s Modified Eagle Medium (Nissui, Tokyo, Japan) supplemented with 10% (*v*/*v*) fetal bovine serum (Thermo Fischer Scientific-gibco), 0.3% (*w*/*v*) sodium bicarbonate (Fujifilm Wako, Osaka, Japan), 100 units/mL penicillin, 100 µg/mL streptomycin, 292 µg/mL L-glutamine, 1× nonessential amino acids solution (Thermo Fischer Scientific-gibco), and 10 ng/mL recombinant mouse IL-3, which was obtained by the baculovirus expression system [42]. After 4–6 weeks of culture, >97% of the cells were identified as c-Kit^+^FcεRIα^+^ MCs by flow cytometry, as described below.

The fibroblast-directed maturation of immature BMMCs toward connective tissue-type MC (CTMC)-like cells was described previously [16,43,44,45]. Briefly, BMMCs were seeded onto the monolayer of Swiss 3T3 fibroblasts (Japanese Cancer Research Resource Bank, Osaka, Japan) and cocultured for appropriate periods (typically 4 days) in the presence of 100 ng/mL mouse SCF (Peprotech, Cranbury, NJ, USA). The cells were trypsinized and reseeded in culture dishes, and adherent fibroblasts and nonadherent MCs were collected, or pure MCs were isolated using CD117 Microbeads and autoMACS Pro Separator (Miltenyi Biotec, Tokyo, Japan). The maturation of BMMCs into CTMC-like cells was verified by staining of their granules with alcian blue and counterstaining with safranin O (Muto Pure Chemicals, Tokyo, Japan), as described previously [44].

Before or after coculture with 3T3 fibroblasts, 10^6^ BMMCs were preloaded for 2 h with 1 µg/mL anti-dinitrophenyl (DNP) IgE (clone SPE-7, Sigma-Aldrich, St. Louis, MO, USA) in Tyrode’s buffer. After removal of the excess antibody, the cells were stimulated with 100 ng/mL human serum albumin (HSA) conjugated with DNP (DNP-HSA; Sigma-Aldrich) as an Ag for 10 min at 37 °C. As required for experiments, the cells were directly activated by 10 μg/mL Compound 48/80 (C48/80; Sigma-Aldrich), an IgE-independent MC secretagogue. The degree of degranulation was determined by measuring the release of β-hexosaminidase (β-HEX), as described previously [43]. The levels of PGD_2_ were determined by ELISA in accordance with the manufacturer’s instructions (PGD_2_-MOX ELISA Kit, Cayman Chemical, Ann Arbor, MI, USA).

### 2.3. Flow Cytometry

BMMCs were stained with fluorochrome-conjugated monoclonal antibodies specific for CD117/c-Kit (clone 2B8, FITC, BD Biosciences-BD Pharmingen, San Jose, CA, USA) and FcεRIα (clone MAR-1, PE, Thermo Fisher Scientific-eBiosciences). Flow cytometry was performed on a BD FACSAria III flow cytometer (BD Biosciences) and analyzed using FlowJo (LLC, Ashland, OR, USA) software.

### 2.4. Quantitative RT-PCR

The reagents and instrument required for quantitative RT-PCR were purchased from Thermo Fischer Scientific. Total RNA was isolated from mouse BMMCs, fibroblasts, splenocytes, or ear skin using TRIzol reagent in accordance with the manufacturer’s instruments. First-strand cDNA synthesis was performed using a High-Capacity cDNA Reverse Transcription Kit. Quantitative PCR was performed with a predesigned primer-probe set (TaqMan Gene Expression Assay) and TaqMan Gene Expression Master Mix on a StepOnePlus real-time PCR system. TaqMan Gene Expression Assays for *Pla2g3* (Mm01191142_m1), *Hpgds* (Mm00479846_m1), *Ptgds* (Mm01330613_m1), *Ptgdr* (Mm00436050_m1), *Hdc* (Mm00456104_m1), *Mcpt4* (Mm00487636_g1), *Mcpt6* (Mm00487645_m1), *Kit* (Mm00445212_m1), and *Ifng* (Mm01168134_m1) were used. Expression levels of the transcripts were normalized to *Gapdh* (Mouse GAPD Endogenous Control) or *Kit* and fold changes were calculated by the ΔΔCt method. In essence, genes expressed in MCs were normalized with *Kit*, while those expressed in fibroblasts were normalized with *Gapdh*, in accordance with our previous paper [16].

### 2.5. Anaphylaxis

IgE-mediated or C48/80-induced anaphylactic responses were examined as described previously [16,45]. Briefly, in a model of passive cutaneous anaphylaxis (PCA), mouse ears were passively sensitized by subcutaneous injection with 30 ng of anti-DNP IgE monoclonal antibody. On the next day, the mice were challenged by intravenous injection of a mixture of 60 µg of DNP-HSA and 1 mg Evans blue (Fujifilm Wako). In a model of IgE-independent anaphylaxis, mice were intradermally administrated with 250 ng of C48/80 followed by an immediate intravenous injection with Evans blue. Vascular permeability in the mice were measured 30 min after the Ag challenge. The ear tissues were collected and lysed, and amounts of the dye were determined. In a model of passive systemic anaphylaxis (PSA), mice were passively sensitized by intravenous injection with 16.5 µg of anti-DNP IgE. On the next day, the mice were challenged intravenously with 500 µg of DNP-HSA. The rectal temperature was measured over time after Ag challenge with an electronic thermometer (Physitemp Instruments, Clifton, NJ, USA).

### 2.6. Dermatitis

For an ICD model, mice were directly challenged with 20 µL of 0.3% (*v*/*v*) 2,4-dinitrofluorobenzene (DNFB; Sigma-Aldrich) in a vehicle of acetone/olive oil (4:1) to the ear (10 µL to each side). For a CHS model, mice were sensitized on the shaved abdomen with 50 µL of 0.5% (*v*/*v*) DNFB. At 5 days after sensitization, mice were challenged with 20 µL of 0.3% (*v*/*v*) DNFB to the ear (10 µL to each side), as described previously [46,47]. Ear thickness was measured before and at 4 h in ICD and at several intervals after hapten challenge in CHS with a micrometer (Mitsutoyo, Kanagawa, Japan).

### 2.7. MC Reconstitution

BMMCs (10^6^) were reconstituted for 6 weeks by intradermal injection into 6-week-old MC-deficient *Kit*^W-sh^ mice [16,30]. The mice were subjected to CHS, as described above. Alternatively, MCs from the base to the tip of the ears from these mice were evaluated histologically by toluidine blue staining, as described below.

### 2.8. Histology

Ear pinnae from mice were fixed with 10% (*v*/*v*) formalin solution and embedded in paraffin, and 4 µm sections were cut and then stained with toluidine blue or hematoxylin and eosin (Merck Millipore, Burlington, MA, USA). For MC quantification, diffuse toluidine blue^+^ cells with no clearly defined cell membrane indicated MCs.

### 2.9. Statistical Analysis

Results are presented as box plots with Tukey whiskers or mean ± SEM. Statistical analysis was performed with Prism 9 (GraphPad, San Diego, CA, USA) software. Two-tailed Mann–Whitney test and ordinary one-way or two-way ANOVA with post hoc Tukey multiple comparisons test were performed as noted in the respective figure legends.

## 3. Results

### 3.1. Generation of MC-Specific Pla2g3-Deficient Mice

**Figure 1:**
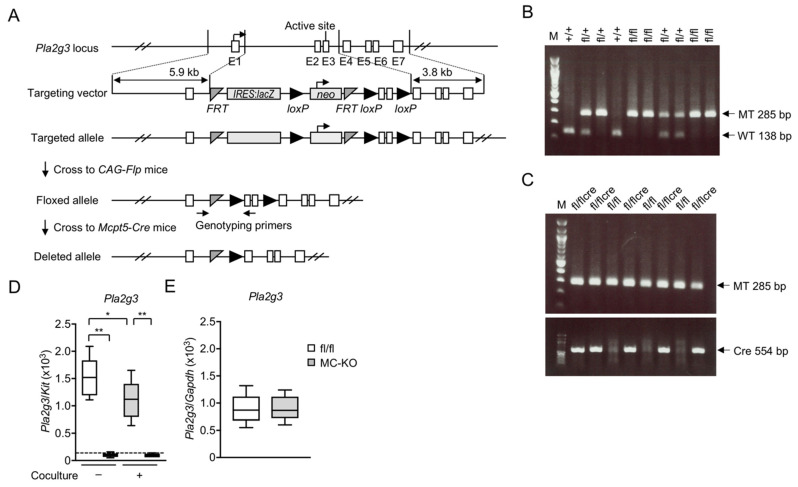
Generation of MC-specific *Pla2g3*-deficient mice. (**A**) A schematic representation of the mouse *Pla2g3* locus, the targeting vector from KOMP, the targeted *Pla2g3* allele with a *lacZ*/*neo* cassette, the floxed *Pla2g3* allele after removal of the *lacZ* cassette by flippase, and the *Pla2g3*-deleted allele after removal of the *neo* cassette by Cre recombinase. The floxed mice were bred with *Mcpt5*-*Cre* mice, and sequences between the two *loxP* sites were removed from the offspring’s genome in an MC-specific manner. Positions of primers for genotyping are marked with arrows. E1–7 (open boxes), *Pla2g3* exons; *FRT* (gray isosceles triangles), sites for flippase; *IRES*, internal ribosome entry site; *lacZ* (gray box), gene encoding β-galactosidase; *neo* (gray box), neomycin phosphotransferase; *loxP* sites (black right triangles), target sites for Cre recombinase. Adapted from www.KOMP.org. (**B**,**C**) A representative genotyping PCR on agarose gels. The amplified PCR products specific for the floxed allele (MT, 285 bp), WT *Pla2g3* allele (WT, 138 bp), and *Mcpt5*-*Cre* allele (Cre, 554 bp) are indicated. (**D**,**E**) Quantitative RT-PCR of *Pla2g3* in BMMCs with (+) or without (−) coculture with 3T3 fibroblasts (*n* = 6) (**D**) and splenocytes (*n* = 8) (**E**) from *Pla2g3*^fl/fl^ (fl/fl) and *Pla2g3*^fl/fl^*Mcpt-5*-*Cre* mice (MC-KO). The dotted line is the threshold for the detection limit. Data are presented as box plots with Tukey whiskers. *, *p* < 0.05; **, *p* < 0.01; one-way ANOVA (**D**); Mann–Whitney test (**E**). Data are pooled from 2 independent experiments, each of which gave similar results.

We successfully generated *Pla2g3*-floxed (*Pla2g3*^fl/fl^) mice, in which the exons encompassing the catalytic domain of the *Pla2g3* gene were replaced with the *IRES*:*lacZ* and *neo* cassettes and flanked by *FRT* or *loxP* sites, for the purpose of conditional inactivation of the gene in a cell- and tissue-specific manner (Figure 1A). To examine the function of *Pla2g3* in MCs, we crossed *Pla2g3*^fl/fl^ mice with *Mcpt5*-*Cre* mice [39], which express Cre recombinase selectively in MCs. Mice with conditional deletion of *Pla2g3* (*Pla2g3*^fl/fl^*Mcpt5*-*Cre*) were screened by genotyping PCR (Figure 1B,C).

BMMCs represent an immature population of MCs [16,43,44,45]. We took advantage of an in vitro system in which immature BMMCs undergo maturation toward mature CTMC-like cells by coculture with Swiss 3T3 fibroblasts [16,43,44,45]. *Mcpt5*, encoding an MC-specific protease, was constantly expressed in BMMCs throughout the coculture period [44]. In *Pla2g3*^fl/fl^*Mcpt5*-*Cre* mice (fl/flcre in Figure 1C), *Pla2g3* expression was largely abrogated in both IL-3-maintained immature BMMCs and cocultured CTMC-like cells (Figure 1D), whereas *Pla2g3* was expressed normally in other cells such as splenocytes (Figure 1E), confirming that Cre-mediated recombination efficiently ablated *Pla2g3* in MCs.

### 3.2. MC-Specific Pla2g3 Ablation Impairs MC Maturation

**Figure 2:**
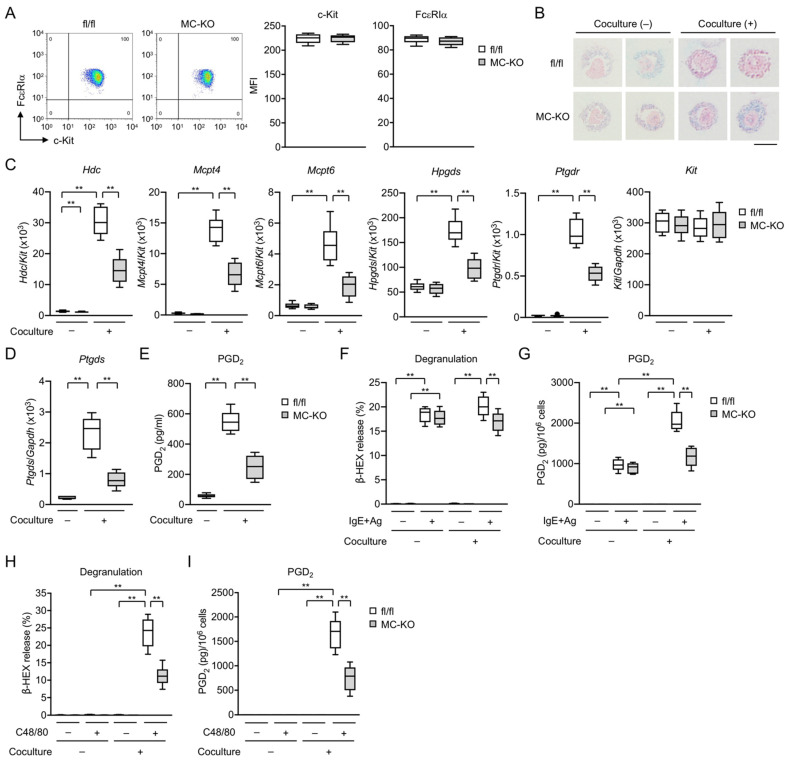
Defective fibroblast-directed maturation of MC-specific *Pla2g3*-null BMMCs. (**A**) FACS analysis of FcεRIα (vertical axis) and c-Kit (horizontal axis) expression on the surface of BMMCs from *Pla2g3*^fl/fl^ (fl/fl) and *Pla2g3*^fl/fl^*Mcpt5*-*Cre* (MC-KO) mice. Representative FACS profiles and mean fluorescence intensities (MFI) of FcεRIα or c-Kit expression on the cell surface (*n* = 6) are shown. (**B**) Alcian blue/safranin staining of BMMCs with (+) or without (−) culture for 4 days with 3T3 fibroblasts. Scale bar, 5 µm. (**C**) mRNA expression levels for *Hdc*, *Mcpt4*, *Mcpt6*, *Hpgds*, *Ptgdr*, and *Kit* in *Pla2g3*^fl/fl^ and *Pla2g3*^fl/fl^*Mcpt5*-*Cre* BMMCs cultured with or without 3T3 fibroblasts (*n* = 6). (**D**) *Ptgds* mRNA levels and (**E**) PGD_2_ production in 3T3 fibroblasts cultured with or without *Pla2g3*^fl/fl^ and *Pla2g3*^fl/fl^*Mcpt5*-*Cre* BMMCs (*n* = 6). (**F**–**I**) *Pla2g3*^fl/fl^ and *Pla2g3*^fl/fl^*Mcpt5*-*Cre* BMMCs cultured with or without 3T3 fibroblasts were sensitized with anti-DNP IgE and then stimulated with DNP-HSA (IgE + Ag), or activated directly with C48/80. Degranulation as assessed by β-HEX release (**F**,**H**) and PGD_2_ production (**G**,**I**) were measured 10 min after MC activation (*n* = 6). Data are compiled from 2 experiments (box plots with Tukey whiskers; **, *p* < 0.01).

BMMCs prepared from global *Pla2g3*^−/−^ mice exhibit impaired fibroblast-driven maturation and thereby IgE-dependent and -independent activation in ex vitro culture [16]. To confirm our findings in global *Pla2g3*^−/−^ mice, we investigated the effects of MC-specific ablation of *Pla2g3* on the maturation and activation of BMMCs before and after coculture with Swiss 3T3 fibroblasts. *Pla2g3* deficiency in IL-3-maintained BMMCs affected neither proliferation (data not shown), cell surface expression of c-Kit and FcεRIα as assessed by flow cytometry (Figure 2A), nor granule staining with alcian blue (Figure 2B). While *Pla2g3*^fl/fl^ BMMCs after coculture contained more safranin-positive granules than did the cells before coculture, *Pla2g3*^fl/fl^*Mcpt5*-*Cre* BMMCs were stained with safranin only weakly even after coculture (Figure 2B), suggesting immaturity of the granules. The maturation of *Pla2g3*^fl/fl^ BMMCs to CTMC-like cells resulted in marked induction of *Hdc* (encoding histidine decarboxylase, a histamine-biosynthetic enzyme), MC-specific proteases *Mcpt4* and *Mcpt6* (encoding chymase and tryptase, respectively), *Hpgds* (encoding H-PGDS, which is responsible for PGD_2_ production in BMMCs but not in fibroblasts), and *Ptgdr* (encoding DP1, a PGD_2_ receptor that provides an MC maturation signal) [16]. The induction of these MC maturation markers was markedly impaired, whereas the constitutive expression of *Kit* was unaffected, in *Pla2g3*^fl/fl^*Mcpt5*-*Cre* BMMCs (Figure 2C). In addition, the induction of *Ptgds* (encoding L-PGDS, which is responsible for PGD_2_ production in fibroblasts but not in BMMCs) (Figure 2D) and PGD_2_ production (Figure 2E) were lower in fibroblasts cocultured with *Pla2g3*^fl/fl^*Mcpt5*-*Cre* BMMCs than in those cocultured with *Pla2g3*^fl/fl^ BMMCs, verifying a bidirectional interaction between MCs and fibroblasts.

After but not before coculture, FcεRI-dependent release of β-HEX (a degranulation marker) following sensitization with DNP-specific IgE and challenge with DNP-HSA as an Ag was significantly lower in *Pla2g3*^fl/fl^*Mcpt5*-*Cre* BMMCs than in *Pla2g3*^fl/fl^ BMMCs (Figure 2F). Because *Hdc* expression was markedly reduced in *Pla2g3*^fl/fl^*Mcpt5*-*Cre* BMMCs after coculture (Figure 2C), it is likely that histamine synthesis and release were concomitantly reduced in these cells, as seen in global *Pla2g3*^−/−^ mice [16]. The maturation of *Pla2g3*^fl/fl^ BMMCs to CTMC-like cells increased IgE-dependent PGD_2_ synthesis (Figure 2G), with a concomitant increase in *Hpgds* expression (Figure 2C). However, the coculture-driven elevation of IgE-mediated PGD_2_ generation occurred only modestly in *Pla2g3*^fl/fl^*Mcpt5*-*Cre* cells (Figure 2G), most likely due to the reduced induction of *Hpgds* (Figure 2C). Although cocultured *Pla2g3*^fl/fl^ CTMC-like cells acquired sensitivity to C48/80, which acts on mouse Mas-related G protein-coupled receptor B2 (Mrgprb2; the ortholog of human MRGPRX2 [48]), C48/80-induced degranulation and PGD_2_ production were markedly lower in cocultured *Pla2g3*^fl/fl^*Mcpt5*-*Cre* cells (Figure 2H,I). Thus, as in the case of global *Pla2g3* deletion [16], MC-specific *Pla2g3* deletion impairs fibroblast-directed maturation and thereby IgE-mediated and even -independent activation of BMMCs in ex vitro culture.

### 3.3. MC-Specific Pla2g3 Ablation Ameliorates MC-Associated Anaphylaxis and Irritant Dermatitis

**Figure 3:**
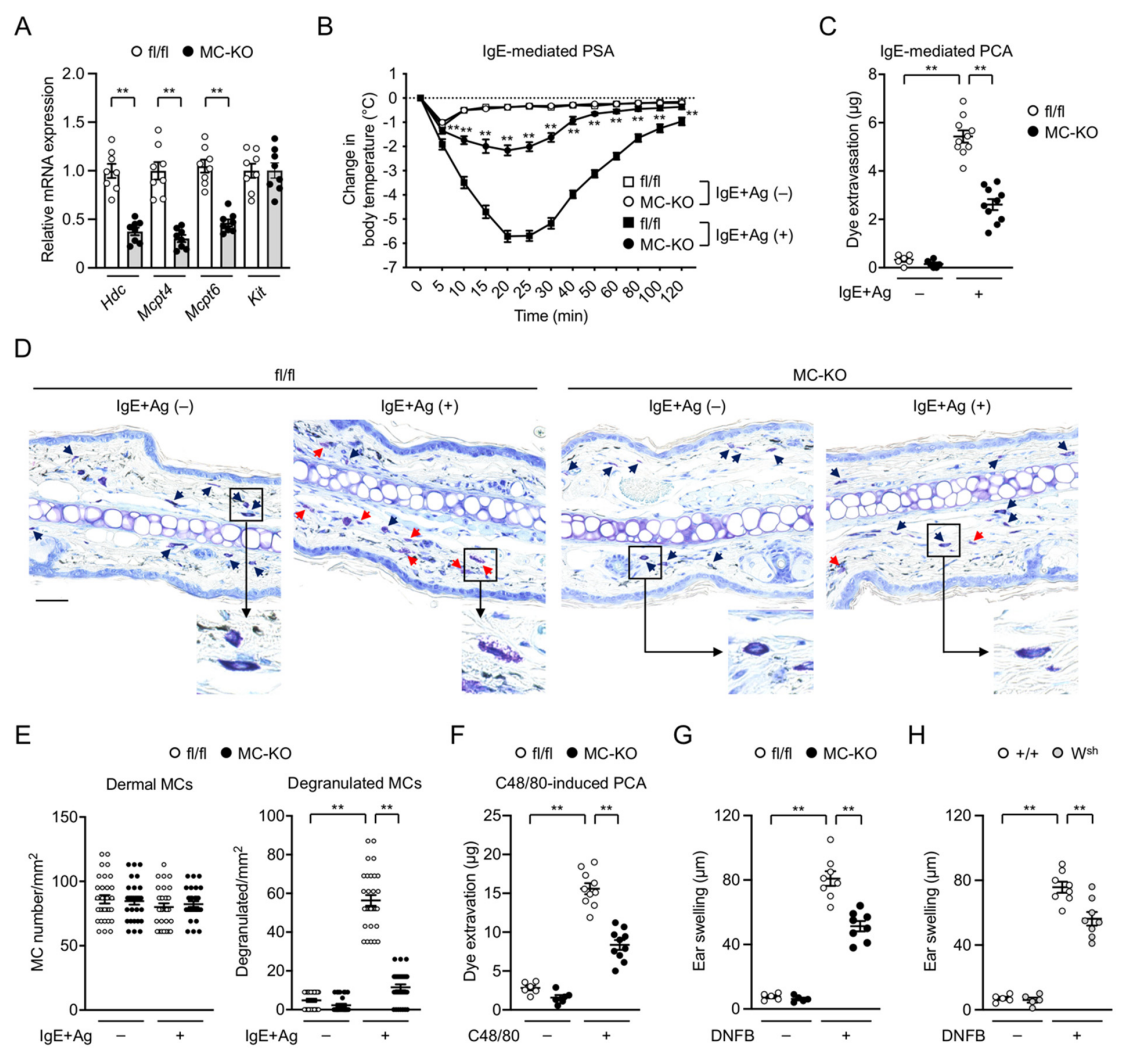
Impaired anaphylaxis and irritant dermatitis in MC-specific *Pla2g3*-deficient mice. (**A**) Expression of *Hdc*, *Mcpt4*, *Mcpt6*, and *Kit* in ear skin of *Pla2g3*^fl/fl^ (fl/fl) and *Pla2g3*^fl/fl^*Mcpt5*-*Cre* (MC-KO) mice (*n* = 8). *Gapdh* was used as a reference gene for normalization. Expression levels of individual genes in fl/fl were regarded as 1. (**B**) IgE-mediated PSA. Changes in rectal temperatures over time after challenge with vehicle (IgE + Ag (−), *n* = 5) or DNP-HSA (IgE + Ag (+), *n* = 8) in *Pla2g3*^fl/fl^ (squares) and *Pla2g3*^fl/fl^*Mcpt5*-*Cre* (circles) mice. **, *p* < 0.01 for *Pla2g3*^fl/fl^*Mcpt5*-*Cre* mice *versus Pla2g3*^fl/fl^ mice. (**C**) IgE-mediated PCA. Dye extravasation 30 min after challenge with vehicle (IgE + Ag (−), *n* = 5) or DNP-HSA (IgE + Ag (+), *n* = 10) in *Pla2g3*^fl/fl^ and *Pla2g3*^fl/fl^*Mcpt5*-*Cre* mice. (**D**) Photomicrographs of representative toluidine blue-stained sections of ear pinnae from *Pla2g3*^fl/fl^ and *Pla2g3*^fl/fl^*Mcpt5*-*Cre* mice 2 min after vehicle or Ag challenge. *Insets* show higher magnification of each boxed area. Arrowheads indicate MCs (resting, black arrows; degranulated, red arrows). Scale bar, 25 µm. (**E**) The number of dermal MCs/mm^2^ after vehicle or Ag challenge. Thirty views for each group (*n* = 5). (**F**) C48/80-induced PCA. Dye extravasation 30 min after administration with vehicle (−, *n* = 5) or C48/80 (+, *n* = 10) in *Pla2g3*^fl/fl^ and *Pla2g3*^fl/fl^*Mcpt5*-*Cre* mice. (**G**,**H**) DNFB-induced ICD. Ear swelling after treatment for 4 h with vehicle (−, *n* = 5) or DNFB (+, *n* = 8) in *Pla2g3*^fl/fl^ and *Pla2g3*^fl/fl^*Mcpt5*-*Cre* mice (**G**) or *Kit*^W-sh^ (W^sh^) and *Kit*^+/+^ (+/+) controls (**H**). Data, mean ± SEM. **, *p* < 0.01; (**A**) two-tailed Mann–Whitney test; (**B**) two-way and (**C**,**E**–**H**) one-way ANOVA. Data are pooled from 2 independent experiments, each of which gave similar results.

We then evaluated the impacts of MC-specific *Pla2g3* deficiency on anaphylactic responses in vivo. In agreement with the ex vivo experiments (Figure 2), the expression levels of *Hdc*, *Mcpt4*, and *Mcpt6* were notably lower, while that of *Kit* was unaffected, in the ear skin of *Pla2g3*^fl/fl^*Mcpt5*-*Cre* mice relative to *Pla2g3*^fl/fl^ mice (Figure 3A), confirming the defective MC maturation by the MC-specific absence of sPLA_2_-III in vivo. To evaluate systemic anaphylaxis, we monitored the changes in body temperature after intravenous injections of Ag-specific IgE followed by that of Ag. *Pla2g3*^fl/fl^ mice showed a severe transient drop in rectal temperature after systemic Ag challenge, whereas this response was markedly impaired in *Pla2g3*^fl/fl^*Mcpt5*-*Cre* mice (Figure 3B). As for local anaphylaxis, mouse ears were injected intradermally with IgE, followed by systemic Ag challenge with Evans blue as an extravasation tracer. Extravasation of the dye in response to Ag challenge was markedly lower in *Pla2g3*^fl/fl^*Mcpt5*-*Cre* mice than in *Pla2g3*^fl/fl^ mice (Figure 3C). Although the ear skin of *Pla2g3*^fl/fl^*Mcpt5*-*Cre* and *Pla2g3*^fl/fl^ mice contained an equivalent number of toluidine blue^+^ MCs, cells showing signs of Ag-induced degranulation were fewer in *Pla2g3*^fl/fl^*Mcpt5*-*Cre* mice than in *Pla2g3*^fl/fl^ mice (Figure 3D,E).

It has recently been shown that ICD requires CTMC activation in a manner dependent on substance P released from nociceptive neurons and MrgprB2 expressed on MCs [49]. MrgprB2 functions as an activating receptor for IgE-independent CTMC degranulation in response to multiple exogenous and endogenous ligands such as C48/80 and substance P [48,49,50,51]. We observed that the IgE-independent, C48/80-induced anaphylactic response was markedly lower in *Pla2g3*^fl/fl^*Mcpt5*-*Cre* mice than in *Pla2g3*^fl/fl^ mice (Figure 3F). Upon ICD, acute ear swelling in response to a single challenge with DNFB was significantly attenuated in *Pla2g3*^fl/fl^*Mcpt5*-*Cre* mice compared with *Pla2g3*^fl/fl^ mice (Figure 3G) as well as in MC-deficient *Kit*^W-sh^ mice compared with *Kit*^+/+^ mice (Figure 3H). Collectively, these results indicate that the ablation of *Pla2g3* specifically in MCs leads to impaired MC maturation, accompanied by reduced MC-associated anaphylaxis and irritant dermatitis, in vivo.

### 3.4. MC-Specific Pla2g3 Ablation Excacerbates CHS

The contribution of MCs to CHS, a delayed-type allergic response that depends on Th1 immunity, remains controversial. Indeed, the use of different MC-deficient animals has suggested a positive immunostimulatory role [52,53,54,55,56], a negative immunomodulatory role [35,57,58,59], or no role [60,61,62] of MCs in several CHS models depending on the experimental conditions used. In this study, to evaluate how MC-derived sPLA_2_-III would contribute to CHS, we subjected MC-specific *Pla2g3*^fl/fl^-deficient mice to a model of DNFB-induced CHS.

**Figure 4:**
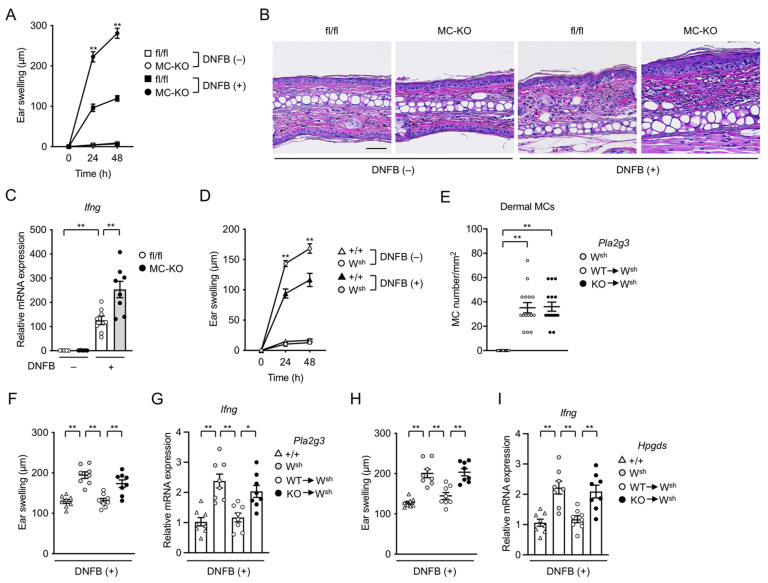
Exacerbated hapten-induced CHS by deficiency of *Pla2g3* or *Hpgds* in MCs. (**A**) Changes in ear thickness over time after second challenge with vehicle (−, *n* = 5) or DNFB (+, *n* = 8) in *Pla2g3*^fl/fl^ (fl/fl, squares) and *Pla2g3*^fl/fl^*Mcpt5*-*Cre* (MC-KO, circles) mice. (**B**) Representative photomicrographs of sections of ear pinnae in *Pla2g3*^fl/fl^ and *Pla2g3*^fl/fl^*Mcpt5*-*Cre* mice sacrificed at 48 h with (+) or without (−) the second DNFB challenge. Scale bar, 25 µm. (**C**) Expression of *Ifng* in ear skin of vehicle (*n* = 5) or DNFB (*n* = 8)-treated *Pla2g3*^fl/fl^ and *Pla2g3*^fl/fl^*Mcpt5*-*Cre* mice. *Gapdh* was used as a reference gene for normalization. Expression level in fl/fl without DNFB treatment was regarded as 1. (**D**) Ear swelling in response to vehicle (*n* = 5) or DNFB (*n* = 8) in sensitized *Kit*^W-sh^ (W^sh^, circles) and *Kit*^+/+^ (+/+, triangles) mice. (**E**) The number of dermal MCs/mm^2^ in ear pinnae of *Kit*^W-sh^ mice that had been reconstituted locally with *Pla2g3*^+/+^ (WT→W^sh^) or *Pla2g3*^−/−^ (KO→W^sh^) BMMCs. Thirty views for each group (*n* = 5). (**F**–**I**) Ear swelling (**F,H**) and *Ifng* expression (**G**,**I**) in response to the second DNFB challenge in ear skin of *Kit*^+/+^ (+/+) and *Kit*^W-sh^ mice that had been reconstituted with or without *Pla2g3* WT and *Pla2g3* KO BMMCs (**F**,**G**) or *Hpgds* WT and *Hpgds* KO BMMCs (**H**,**I**) (*n* = 8). In (**G**,**H**), expression level of *Ifng* (normalized to *Gapdh*) in +/+ was regarded as 1. Data, mean ± SEM; *, *p* < 0.05; **, *p* < 0.01; two-way (**A**,**D**) and one-way (**E**–**I**) ANOVA. Data are pooled from 2 independent experiments performed, each of which gave similar results.

After the second challenge (elicitation) with DNFB, ear swelling was markedly increased in *Pla2g3*^fl/fl^*Mcpt5*-*Cre* mice compared to their *Pla2g3*^fl/fl^ counterparts (Figure 4A). Histological examination of tissue sections revealed that DNFB-induced epidermal and dermal hyperplasia and immune cell recruitment were considerably greater in *Pla2g3*^fl/fl^*Mcpt5*-*Cre* mice than in *Pla2g3*^fl/fl^ mice (Figure 4B). Consistent with the dependence of CHS on IFN-γ-producing CD8^+^ T cells [63], the DNFB-induced induction of *Ifng* was higher in the ear of *Pla2g3*^fl/fl^*Mcpt5*-*Cre* mice than in that of *Pla2g3*^fl/fl^ mice (Figure 4C).

In agreement with previous studies demonstrating the exacerbation of DNFB-induced CHS in MC-deficient *Kit*^W-sh^ mice [55,57,59], *Kit*^W-sh^ mice exhibited greater ear swelling than did MC-sufficient *Kit*^+/+^ mice in our CHS settings (Figure 4D). When *Kit*^W-sh^ mice had been intradermally engrafted with BMMCs from global *Pla2g3*^−/−^ mice or those from WT littermates, the numbers of reconstituted MCs in the ear skin were similar between the two groups (Figure 4E). After reconstitution with WT BMMCs, DNFB-increased ear swelling and *Ifng* expression in *Kit*^W-sh^ mice were reduced to levels similar to those in *Kit*^+/+^ mice, whereas these ameliorating effects were scarcely seen in replicate *Kit*^W-sh^ mice reconstituted with *Pla2g3*^−/−^ BMMCs (Figure 4F,G). Thus, our present study using two types of MC-specific *Pla2g3* deletion models indicates that *Pla2g3* in MCs can substantially limit the magnitude of CHS responses.

We have previously shown that sPLA_2_-III secreted from MCs regulates the spatiotemporal mobilization of distinct PGD_2_ pools in tissue microenvironments; it is directly coupled with L-PGDS in adjacent fibroblasts to generate a pool of PGD_2_ that participates in MC maturation, and indirectly affects H-PGDS-driven production of another pool of PGD_2_ in MCs that counteracts the anaphylactic response [16]. PGD_2_ can suppress inflammation by limiting neutrophil infiltration, dendritic cell activation, or other mechanisms [24,25,26,27,64]. To assess the possibility that the increased CHS by MC-specific depletion of *Pla2g3* might be due to the reduced generation of PGD_2_, we tested the CHS response in *Kit*^W-sh^ mice engrafted with BMMCs from *Hpgds*-deficient mice. Although a similar number of MCs was present in the ear of *Kit*^W-sh^ mice that had been reconstituted with *Hpgds*-sufficient or -deficient BMMCs (data not shown), *Kit*^W-sh^ mice transferred with *Hpgds*^−/−^ BMMCs, like those transferred with *Pla2g3*^−/−^ BMMCs (Figure 4F,G), displayed more severe DNFB-induced ear swelling and *Ifng* induction than replicate *Kit*^W-sh^ mice transferred with WT BMMCs (Figure 4H,I). These results suggest that MC-derived sPLA_2_-III limits the CHS response at least in part by a mechanism that depends on the promotion of MC maturation and thereby H-PGDS-driven production of anti-inflammatory PGD_2_.

## 4. Discussion

Global *Pla2g3*^−/−^ mice display reduced anaphylactic responses, which could be attributed to impairment of MC maturation [16]. sPLA_2_-III, which is likely to be secreted from MCs, acts on adjacent fibroblasts as a paracrine factor to promote the biosynthesis of PGD_2_, an AA-derived lipid mediator that in turn acts on its receptor DP1 on MCs to coordinate proper MC maturation. In this study, using a conditional KO strain in which the *Pla2g3* gene is disrupted specifically in MCs under the *Mcpt5* promoter, we have provided compelling evidence that MC-derived sPLA_2_-III is indeed a critical regulator of MC maturation. Although several studies have reported that *Mcpt5* is not expressed in all MC populations and recommended to use *Cpa3*-*Cre* mice rather than *Mcpt5*-*Cre* mice [39,65,66], our present study shows that, as in the case of global *Pla2g3* deletion [16], MC-specific *Pla2g3* deletion in *Pla2g3*^fl/fl^*Mcpt5*-*Cre* mice leads to reduced upregulation of several MC maturation markers, accompanied by decreased degranulation, PGD_2_ generation, C48/80 sensitivity, and safranin staining, in BMMCs after coculture with fibroblasts ex vivo, as well as reduced IgE-dependent and -independent systemic and local anaphylactic responses in vivo. These results confirm the crucial role of MC-expressed sPLA_2_-III in the proper maturation of MCs and underline the future use of *Pla2g3*^fl/fl^ mice to clarify the cell/tissue-specific functions of sPLA_2_-III in various pathophysiological circumstances.

Our present study has also provided additional insight into the role of MCs in CHS. On the basis of recent research, the roles of MCs in the sensitization and elicitation phases of CHS have been considered as follows: First, during the sensitization phase (which is equivalent to the condition of ICD), MCs are activated directly or indirectly by haptens to release a diverse spectrum of mediators, including histamine and TNF-α, which induce vasodilatation and neutrophil recruitment [53,55]. Consistently, ICD-induced ear swelling, which depends on Mrgprb2 activation [49], is substantially reduced in MC-specific *Pla2g3*-deficient mice. Interactions between MCs and dendritic cells (DCs) by direct contact or MC-secreted TNF-α can amplify DC migration into the draining lymph nodes, where DCs prime naïve T cells to become effector T cells via Ag presentation [67]. Second, during moderate CHS responses, MCs amplify ear swelling, epidermal hyperplasia, and recruitment of neutrophils and CD8^+^ T cells through releasing TNF-α [34]. Third, during more severe CHS responses, MCs represent an early source of IL-10, which amplifies subsequent recruitment of regulatory T (T_reg_) cells and limits ear swelling and epidermal hyperplasia [35,57]. Additionally, MCs can migrate into lymphoid organs, where they produce IL-2, which contributes to maintaining T_reg_ cells and thereby ameliorating CHS [58]. The CHS model employed in the present study fits with the third case, where MCs limit the severity of CHS inflammation. Importantly, the absence of sPLA_2_-III in MCs dampens the suppressive effect of these cells on ear swelling and *Ifng* induction in CHS, revealing a novel role of this particular sPLA_2_ in a specific pathological event. This function appears to depend at least partly on the sPLA_2_-III-driven, indirect mobilization of PGD_2_ by MCs, where MC-derived sPLA_2_-III is coupled with L-PGDS-dependent production of the first pool of PGD_2_ in fibroblasts, which then facilitates the proper maturation of MCs and thereby H-PGDS-dependent production of the second pool of PGD_2_ by these cells. Indeed, *Hpgds* deficiency in MCs also abrogates the suppressive effect of MCs on CHS, highlighting that, in addition to the cytokines IL-2 and IL-10 [33,35,57,58], the lipid mediator PGD_2_ acts as another negative modulator of the CHS responses. In support of this view, mice lacking DP1 (*Ptgdr*^−/−^) also display an exacerbation of CHS by affecting the expression of IL-10 in DCs [26].

Individual sPLA_2_s exhibit unique tissue or cellular distributions and enzymatic properties and exert their specific functions by producing lipid mediators; by altering membrane phospholipid composition; by degrading foreign phospholipids from microorganisms or diet; or by modifying extracellular noncellular lipid compounds such as lipoproteins, pulmonary surfactant, or extracellular vesicles in response to given microenvironmental cues [68,69,70,71]. Currently, two sPLA_2_ isoforms have been reported to participate in the regulation of CHS. sPLA_2_-IID, which is expressed in lymph node DCs, protects against CHS by putting a brake on Th1 immunity through mobilization of ω3 polyunsaturated fatty acids and their anti-inflammatory/proresolving metabolites [46]. sPLA_2_-IIF, which is expressed in keratinocytes, exacerbates CHS by facilitating epidermal hyperplasia through the generation of a unique lysophospholipid [47]. The present study showing that sPLA_2_-III in MCs plays a role in limiting CHS by mobilizing anti-inflammatory PGD_2_ in a feed-forward loop of MC–fibroblast communication represents the third example of the sPLA_2_-mediated regulation of CHS, further highlighting the diverse functions of individual sPLA_2_ isoforms in mobilizing distinct lipids in different cells and tissues.

## Data Availability

The data presented in this study are available on request from the corresponding author.

## References

[1] Galli S.J., Grimbaldeston M., Tsai M. (2008). Immunomodulatory mast cells: Negative, as well as positive, regulators of immunity. Nat. Rev. Immunol..

[2] Galli S.J., Tsai M. (2012). IgE and mast cells in allergic disease. Nat. Med..

[3] Galli S.J., Gaudenzio N., Tsai M. (2020). Mast cells in inflammation and disease: Recent progress and ongoing concerns. Annu. Rev. Immunol..

[4] Boyce J.A. (2007). Mast cells and eicosanoid mediators: A system of reciprocal paracrine and autocrine regulation. Immunol. Rev..

[5] Metcalfe D.D., Baram D., Mekori Y.A. (1997). Mast cells. Physiol. Rev..

[6] Olivera A., Beaven M.A., Metcalfe D.D. (2018). Mast cells signal their importance in health and disease. J. Allergy Clin. Immunol..

[7] Gilfillan A.M., Beaven M.A. (2011). Regulation of mast cell responses in health and disease. Crit. Rev. Immunol..

[8] Gurish M.F., Austen K.F. (2012). Developmental origin and functional specialization of mast cell subsets. Immunity.

[9] Hallgren J., Gurish M.F. (2011). Mast cell progenitor trafficking and maturation. Adv. Exp. Med. Biol..

[10] Kitamura Y. (1989). Heterogeneity of mast cells and phenotypic change between subpopulations. Annu. Rev. Immunol..

[11] Gentek R., Ghigo C., Hoeffel G., Bulle M.J., Msallam R., Gautier G., Launay P., Chen J., Ginhoux F., Bajenoff M. (2018). Hemogenic endothelial fate mapping reveals dual developmental origin of mast cells. Immunity.

[12] Li Z., Liu S., Xu J., Zhang X., Han D., Liu J., Xia M., Yi L., Shen Q., Xu S. (2018). Adult connective tissue-resident mast cells originate from late erythro-myeloid progenitors. Immunity.

[13] Dahlin J.S., Hallgren J. (2015). Mast cell progenitors: Origin, development and migration to tissues. Mol. Immunol..

[14] Kitamura Y., Oboki K., Ito A. (2007). Development of mast cells. Proc. Jpn. Acad. Ser. B Phys. Biol. Sci..

[15] Hallgren J., Gurish M.F. (2007). Pathways of murine mast cell development and trafficking: Tracking the roots and routes of the mast cell. Immunol. Rev..

[16] Taketomi Y., Ueno N., Kojima T., Sato H., Murase R., Yamamoto K., Tanaka S., Sakanaka M., Nakamura M., Nishito Y. (2013). Mast cell maturation is driven via a group III phospholipase A_2_-prostaglandin D_2_-DP1 receptor paracrine axis. Nat. Immunol..

[17] Murakami M. (2017). Lipoquality control by phospholipase A_2_ enzymes. Proc. Jpn. Acad. Ser. B Phys. Biol. Sci..

[18] Murakami M., Taketomi Y., Miki Y., Sato H., Hirabayashi T., Yamamoto K. (2011). Recent progress in phospholipase A_2_ research: From cells to animals to humans. Prog. Lipid Res..

[19] Fujishima H., Sanchez Mejia R.O., Bingham C.O., Lam B.K., Sapirstein A., Bonventre J.V., Austen K.F., Arm J.P. (1999). Cytosolic phospholipase A_2_ is essential for both the immediate and the delayed phases of eicosanoid generation in mouse bone marrow-derived mast cells. Proc. Natl. Acad. Sci. USA.

[20] Nakatani N., Uozumi N., Kume K., Murakami M., Kudo I., Shimizu T. (2000). Role of cytosolic phospholipase A_2_ in the production of lipid mediators and histamine release in mouse bone-marrow-derived mast cells. Biochem. J..

[21] Murakami M., Taketomi Y. (2015). Secreted phospholipase A_2_ and mast cells. Allergol. Int..

[22] Matsuoka T., Hirata M., Tanaka H., Takahashi Y., Murata T., Kabashima K., Sugimoto Y., Kobayashi T., Ushikubi F., Aze Y. (2000). Prostaglandin D_2_ as a mediator of allergic asthma. Science.

[23] Spik I., Brenuchon C., Angeli V., Staumont D., Fleury S., Capron M., Trottein F., Dombrowicz D. (2005). Activation of the prostaglandin D_2_ receptor DP2/CRTH2 increases allergic inflammation in mouse. J. Immunol..

[24] Trivedi S.G., Newson J., Rajakariar R., Jacques T.S., Hannon R., Kanaoka Y., Eguchi N., Colville-Nash P., Gilroy D.W. (2006). Essential role for hematopoietic prostaglandin D_2_ synthase in the control of delayed type hypersensitivity. Proc. Natl. Acad. Sci. USA.

[25] Hammad H., Kool M., Soullie T., Narumiya S., Trottein F., Hoogsteden H.C., Lambrecht B.N. (2007). Activation of the D prostanoid 1 receptor suppresses asthma by modulation of lung dendritic cell function and induction of regulatory T cells. J. Exp. Med..

[26] Yamamoto Y., Otani S., Hirai H., Nagata K., Aritake K., Urade Y., Narumiya S., Yokozeki H., Nakamura M., Satoh T. (2011). Dual functions of prostaglandin D_2_ in murine contact hypersensitivity via DP and CRTH2. Am. J. Pathol..

[27] Nakamura T., Maeda S., Horiguchi K., Maehara T., Aritake K., Choi B.I., Iwakura Y., Urade Y., Murata T. (2015). PGD_2_ deficiency exacerbates food antigen-induced mast cell hyperplasia. Nat. Commun..

[28] Lambeau G., Gelb M.H. (2008). Biochemistry and physiology of mammalian secreted phospholipases A_2_. Annu. Rev. Biochem..

[29] Valentin E., Ghomashchi F., Gelb M.H., Lazdunski M., Lambeau G. (2000). Novel human secreted phospholipase A_2_ with homology to the group III bee venom enzyme. J. Biol. Chem..

[30] Grimbaldeston M.A., Chen C.C., Piliponsky A.M., Tsai M., Tam S.Y., Galli S.J. (2005). Mast cell-deficient *W-sash c-kit* mutant *Kit^W-sh/W-sh^* mice as a model for investigating mast cell biology in vivo. Am. J. Pathol..

[31] Nigrovic P.A., Gray D.H., Jones T., Hallgren J., Kuo F.C., Chaletzky B., Gurish M., Mathis D., Benoist C., Lee D.M. (2008). Genetic inversion in mast cell-deficient W^sh^ mice interrupts *corin* and manifests as hematopoietic and cardiac aberrancy. Am. J. Pathol..

[32] Reber L.L., Marichal T., Galli S.J. (2012). New models for analyzing mast cell functions in vivo. Trends Immunol..

[33] Gaudenzio N., Marichal T., Galli S.J., Reber L.L. (2018). Genetic and imaging approaches reveal pro-inflammatory and immunoregulatory roles of mast cells in contact hypersensitivity. Front. Immunol..

[34] Dudeck J., Ghouse S.M., Lehmann C.H., Hoppe A., Schubert N., Nedospasov S.A., Dudziak D., Dudeck A. (2015). Mast-cell-derived TNF amplifies CD8^+^ dendritic cell functionality and CD8^+^ T cell priming. Cell Rep..

[35] Reber L.L., Sibilano R., Starkl P., Roers A., Grimbaldeston M.A., Tsai M., Gaudenzio N., Galli S.J. (2017). Imaging protective mast cells in living mice during severe contact hypersensitivity. JCI Insight.

[36] Li Y., Liu B., Harmacek L., Long Z., Liang J., Lukin K., Leach S.M., O’Connor B., Gerber A.N., Hagman J. (2018). The transcription factors GATA2 and microphthalmia-associated transcription factor regulate *Hdc* gene expression in mast cells and are required for IgE/mast cell-mediated anaphylaxis. J. Allergy Clin. Immunol..

[37] Skarnes W.C., Rosen B., West A.P., Koutsourakis M., Bushell W., Iyer V., Mujica A.O., Thomas M., Harrow J., Cox T. (2011). A conditional knockout resource for the genome-wide study of mouse gene function. Nature.

[38] Mishina M., Sakimura K. (2007). Conditional gene targeting on the pure C57BL/6 genetic background. Neurosci. Res..

[39] Scholten J., Hartmann K., Gerbaulet A., Krieg T., Muller W., Testa G., Roers A. (2008). Mast cell-specific Cre/loxP-mediated recombination in vivo. Transgenic Res..

[40] Sato H., Taketomi Y., Isogai Y., Miki Y., Yamamoto K., Masuda S., Hosono T., Arata S., Ishikawa Y., Ishii T. (2010). Group III secreted phospholipase A_2_ regulates epididymal sperm maturation and fertility in mice. J. Clin. Invest..

[41] Mohri I., Taniike M., Taniguchi H., Kanekiyo T., Aritake K., Inui T., Fukumoto N., Eguchi N., Kushi A., Sasai H. (2006). Prostaglandin D_2_-mediated microglia/astrocyte interaction enhances astrogliosis and demyelination in *twitcher*. J. Neurosci..

[42] Murakami M., Matsumoto R., Austen K.F., Arm J.P. (1994). Prostaglandin endoperoxide synthase-1 and -2 couple to different transmembrane stimuli to generate prostaglandin D_2_ in mouse bone marrow-derived mast cells. J. Biol. Chem..

[43] Ogasawara T., Murakami M., Suzuki-Nishimura T., Uchida M.K., Kudo I. (1997). Mouse bone marrow-derived mast cells undergo exocytosis, prostanoid generation, and cytokine expression in response to G protein-activating polybasic compounds after coculture with fibroblasts in the presence of *c-kit* ligand. J. Immunol..

[44] Taketomi Y., Sugiki T., Saito T., Ishii S., Hisada M., Suzuki-Nishimura T., Uchida M.K., Moon T.C., Chang H.W., Natori Y. (2003). Identification of NDRG1 as an early inducible gene during in vitro maturation of cultured mast cells. Biochem. Biophys. Res. Commun..

[45] Taketomi Y., Sunaga K., Tanaka S., Nakamura M., Arata S., Okuda T., Moon T.C., Chang H.W., Sugimoto Y., Kokame K. (2007). Impaired mast cell maturation and degranulation and attenuated allergic responses in *Ndrg1*-deficient mice. J. Immunol..

[46] Miki Y., Yamamoto K., Taketomi Y., Sato H., Shimo K., Kobayashi T., Ishikawa Y., Ishii T., Nakanishi H., Ikeda K. (2013). Lymphoid tissue phospholipase A_2_ group IID resolves contact hypersensitivity by driving antiinflammatory lipid mediators. J. Exp. Med..

[47] Yamamoto K., Miki Y., Sato M., Taketomi Y., Nishito Y., Taya C., Muramatsu K., Ikeda K., Nakanishi H., Taguchi R. (2015). The role of group IIF-secreted phospholipase A_2_ in epidermal homeostasis and hyperplasia. J. Exp. Med..

[48] McNeil B.D., Pundir P., Meeker S., Han L., Undem B.J., Kulka M., Dong X. (2015). Identification of a mast-cell-specific receptor crucial for pseudo-allergic drug reactions. Nature.

[49] Zhang S., Edwards T.N., Chaudhri V.K., Wu J., Cohen J.A., Hirai T., Rittenhouse N., Schmitz E.G., Zhou P.Y., McNeil B.D. (2021). Nonpeptidergic neurons suppress mast cells via glutamate to maintain skin homeostasis. Cell.

[50] Serhan C.N., Levy B.D. (2018). Resolvins in inflammation: Emergence of the pro-resolving superfamily of mediators. J. Clin. Invest..

[51] Green D.P., Limjunyawong N., Gour N., Pundir P., Dong X. (2019). A mast-cell-specific receptor mediates neurogenic inflammation and pain. Neuron.

[52] Askenase P.W., Van Loveren H., Kraeuter-Kops S., Ron Y., Meade R., Theoharides T.C., Nordlund J.J., Scovern H., Gerhson M.D., Ptak W. (1983). Defective elicitation of delayed-type hypersensitivity in W/W^v^ and SI/SI^d^ mast cell-deficient mice. J. Immunol..

[53] Biedermann T., Kneilling M., Mailhammer R., Maier K., Sander C.A., Kollias G., Kunkel S.L., Hultner L., Rocken M. (2000). Mast cells control neutrophil recruitment during T cell-mediated delayed-type hypersensitivity reactions through tumor necrosis factor and macrophage inflammatory protein 2. J. Exp. Med..

[54] Bryce P.J., Miller M.L., Miyajima I., Tsai M., Galli S.J., Oettgen H.C. (2004). Immune sensitization in the skin is enhanced by antigen-independent effects of IgE. Immunity.

[55] Dudeck A., Dudeck J., Scholten J., Petzold A., Surianarayanan S., Kohler A., Peschke K., Vohringer D., Waskow C., Krieg T. (2011). Mast cells are key promoters of contact allergy that mediate the adjuvant effects of haptens. Immunity.

[56] Otsuka A., Kubo M., Honda T., Egawa G., Nakajima S., Tanizaki H., Kim B., Matsuoka S., Watanabe T., Nakae S. (2011). Requirement of interaction between mast cells and skin dendritic cells to establish contact hypersensitivity. PLoS ONE.

[57] Grimbaldeston M.A., Nakae S., Kalesnikoff J., Tsai M., Galli S.J. (2007). Mast cell-derived interleukin 10 limits skin pathology in contact dermatitis and chronic irradiation with ultraviolet B. Nat. Immunol..

[58] Hershko A.Y., Suzuki R., Charles N., Alvarez-Errico D., Sargent J.L., Laurence A., Rivera J. (2011). Mast cell interleukin-2 production contributes to suppression of chronic allergic dermatitis. Immunity.

[59] Gimenez-Rivera V.A., Siebenhaar F., Zimmermann C., Siiskonen H., Metz M., Maurer M. (2016). Mast cells limit the exacerbation of chronic allergic contact dermatitis in response to repeated allergen exposure. J. Immunol..

[60] Galli S.J., Hammel I. (1984). Unequivocal delayed hypersensitivity in mast cell-deficient and beige mice. Science.

[61] Mekori Y.A., Galli S.J. (1985). Undiminished immunologic tolerance to contact sensitivity in mast cell-deficient W/W^v^ and Sl/Sl^d^ mice. J. Immunol..

[62] Mekori Y.A., Chang J.C., Wershil B.K., Galli S.J. (1987). Studies of the role of mast cells in contact sensitivity responses. Passive transfer of the reaction into mast cell-deficient mice locally reconstituted with cultured mast cells: Effect of reserpine on transfer of the reaction with DNP-specific cloned T cells. Cell. Immunol..

[63] Xu H., DiIulio N.A., Fairchild R.L. (1996). T cell populations primed by hapten sensitization in contact sensitivity are distinguished by polarized patterns of cytokine production: Interferon gamma-producing (Tc1) effector CD8^+^ T cells and interleukin (Il) 4/Il-10-producing (Th2) negative regulatory CD4^+^ T cells. J. Exp. Med..

[64] Levy B.D., Clish C.B., Schmidt B., Gronert K., Serhan C.N. (2001). Lipid mediator class switching during acute inflammation: Signals in resolution. Nat. Immunol..

[65] Wei Y., Chhiba K.D., Zhang F., Ye X., Wang L., Zhang L., Robida P.A., Moreno-Vinasco L., Schnaar R.L., Roers A. (2018). Mast cell-specific expression of human Siglec-8 in conditional knock-in mice. Int. J. Mol. Sci..

[66] Lilla J.N., Chen C.C., Mukai K., BenBarak M.J., Franco C.B., Kalesnikoff J., Yu M., Tsai M., Piliponsky A.M., Galli S.J. (2011). Reduced mast cell and basophil numbers and function in Cpa3-Cre; Mcl-1^fl/fl^ mice. Blood.

[67] Suto H., Nakae S., Kakurai M., Sedgwick J.D., Tsai M., Galli S.J. (2006). Mast cell-associated TNF promotes dendritic cell migration. J. Immunol..

[68] Murakami M., Sato H., Miki Y., Yamamoto K., Taketomi Y. (2015). A new era of secreted phospholipase A_2_. J. Lipid Res..

[69] Murakami M., Yamamoto K., Miki Y., Murase R., Sato H., Taketomi Y. (2016). The roles of the secreted phospholipase A_2_ gene family in immunology. Adv. Immunol..

[70] Murakami M., Miki Y., Sato H., Murase R., Taketomi Y., Yamamoto K. (2019). Group IID, IIE, IIF and III secreted phospholipase A_2_s. Biochim. Biophys. Acta Mol. Cell Biol. Lipids.

[71] Murakami M., Sato H., Taketomi Y. (2020). Updating phospholipase A_2_ biology. Biomolecules.

